# A new insight into FEIA

**DOI:** 10.1186/1710-1492-7-S2-A32

**Published:** 2011-11-14

**Authors:** Jennifer Yan Fei Chen, Jason Kihyuk Lee

**Affiliations:** 1 University of Toronto, Toronto, Ontario, Canada; 2 Division of Allergy and Clinical Immunology, St. Michael’s Hospital, Toronto, Ontario, M5B 1W8, Canada

## 

Two cases of patients with food and exercise-induced anaphylaxis (FEIA) with confirmed allergies to oral allergy syndrome are herein presented. Patient A had food anaphylaxis to fresh coriander and tomato and Patient B to fresh celery. These food allergens have structural antigenic similarity to that of birch and/or grass. Both patients’ allergies were confirmed by fresh prick-to-prick tests. In both cases, strenuous exercise before the reaction was the only cofactor and the patients had absolutely no symptoms with the offending foods outside of exercise. The exercise had likely lowered the threshold for their reactions. The current literature propose that in FEIA, there is increased GI permeability, leading to enhanced allergen absorption [1]. However, van Nieuvenhoven *et al* found that intestinal permeability actually decreases with exercise [2]. In fact, Bi and Triadafilopoulos noted in their review that strenuous exercise delays gastric emptying of liquids and solids and inhibits gastric acid production [3]. These studies have led us to propose of a novel paradigm for the mechanism of FEIA. The general inhibitory effects of exercise on the GI tract decrease the digestion of oral allergens, thus leaving the allergens more structurally intact and thereby allowing continued systemic absorption of the allergen. This mechanism is supported by Untersmayr and Jensen-Jarolim’s findings on the increased risk of labile food allergy induction with the use of antacid medications [4]. We propose the decrease in gastric acid in exercise as a more biologically plausible hypothesis of the mechanism of FEIA to oral allergens foods.
